# Predicting fractures using trabecular patterns on panoramic radiographs

**DOI:** 10.1007/s00784-017-2122-2

**Published:** 2017-06-01

**Authors:** Wil Geraets, Grethe Jonasson, Magnus Hakeberg

**Affiliations:** 1 Department of Oral and Maxillofacial Radiology, Academic Centre for Dentistry Amsterdam (ACTA), Gustav Mahlerlaan 3004, 1081 LA Amsterdam, The Netherlands; 2 Research & Development Unit in Southern Älvsborg County, Sven Eriksonplatsen 4, 50338 Borås, Sweden; 3 Department of Behavioral and Community Dentistry, Institute of Odontology, University of Gothenburg, Medicinaregatan 12E, Gothenburg, Sweden

**Keywords:** Fracture prediction, Panoramic radiographs, Trabecular pattern, Observer score, ROC analysis

## Abstract

**Objectives:**

The observer score of the trabecular pattern on panoramic radiographs is known to be a strong predictor of bone fractures. The aim of this study was to enhance the predictive power of panoramic radiographs by means of texture analysis methods.

**Material and methods:**

The study followed 304 postmenopausal women during 26 years. At the beginning of the study, panoramic radiographs were obtained. One observer assessed the trabecular pattern in the premolar region as dense, sparse, or alternating dense and sparse. In addition, on each radiograph, a region of interest was selected in the molar/premolar region and analyzed with texture analysis procedures. During 26 years of follow-up, 115 women suffered a fracture of the hip, spine, leg, or arm. Logistic regression was applied to test the predictive power of various variables with respect to fractures.

**Results:**

Of all variables, the observer score of the trabecular pattern correlated strongest with the occurrence of fractures. By itself, the score yielded an ROC curve with an area of 0.80 under the curve. Combining the observer score with the texture analysis features increased the area under the ROC curve to 0.85.

**Conclusions:**

The trabecular pattern on panoramic radiographs provides a strong predictor of fractures, at least for postmenopausal women. The assessment by an observer combined with texture analysis procedures yields a predictive power that parallels best known predictions in literature.

**Clinical relevance:**

This study illustrates that panoramic radiographs are state of the art predictors of postcranial fractures.

## Introduction

The greatest complication of bone disease, especially osteoporosis, is the occurrence of fractures. Some 40% of Caucasian women aged 50 years or more experience a fracture of the hip, spine, or wrist during their life. Fractures of the wrist are the most common, but fractures of the hip are the most serious in terms of mortality, morbidity, and cost [1, 2].

The lifetime risk for a hip fracture lies between 14 and 23% among Caucasian women in Europe and the USA and is likely to increase as mortality for other conditions declines [1, 3]. Worldwide, there is substantial variation in hip fracture incidence between populations. Even in Europe, the risk for hip fracture varies about three-fold between countries [3].

The WHO defined osteoporosis as a systemic skeletal disease characterized by reduced bone mineral density (BMD) and microarchitectural deterioration of bone tissue leading to increased risk of fractures. The organization recommended to use BMD measurements to diagnose osteoporosis [4]. The prediction of osteoporosis and the prediction of fractures are related subjects, but different nevertheless. The risk of fractures is high when BMD is low, but it is by no means negligible when BMD is normal [5]. Therefore, the majority of fractures occur in non-osteoporotic subjects, and BMD measurements are not recommended for population screening [5–9]. Other risk factors for fractures include age, previous fractures, body weight, and body mass index (BMI) [5, 9–11]. A fracture of the hip or spine more than doubles the risk of a subsequent fracture [5, 11].

Fracture prediction uses statistical models to identify people at high risk of fractures. The most common way of measuring the discriminative power of a prediction model is plotting a receiver operating characteristic (ROC) curve [12, 13]. The predictive power of the model then is given by the area under the curve (AUC) [13]. The WHO developed a Fracture Risk Assessment tool (FRAX), to assess the fracture risk based on the most relevant risk factors such as age, sex, weight, height, previous fracture, parent hip fracture, current smoking, glucocorticoid use, rheumatoid arthritis, alcohol use, and femoral neck BMD [9]. With respect to the predic-tion of major osteoporotic fractures, AUC values up to 0.69 have been reported for FRAX [14, 15]. Alternative tools such as the Osteoporosis Self assessment Tool (OST), the Simple Calculated Osteoporosis Risk Estimation (SCORE), and the FRACTURE Index were developed. OST is based entirely on gender, age, and weight whereas SCORE also involves race, rheumatoid arthritis, estrogen therapy, and fracture history. The FRACTURE Index involves gender, age, weight, BMD, fracture history, and maternal fracture history. Most of these tools performed as accurately as FRAX [16–19]. However, some outperformed FRAX with an AUC value of 0.76 or 0.77 [18–20]. An Australian study reported an im-pressive AUC of 0.84 using the Garvan algorithm which is based on sex, age, BMD, fall history, and fracture history [21]. Considering that an AUC of 1.00 represents perfect prediction, it seems that at the present state of the art, there is still room for improvement. However, since an element of chance is involved in the occurrence of fractures, perfect prediction is not attainable and it remains to be seen how much the prediction of osteoporotic fractures can be improved.

Moreover, fracture prediction proves to be an elusive issue. An elaborate Norwegian validation study of the Garvan algorithm gave an AUC of only 0.62 [22]. In 2014, an alarming report on fracture prediction was published [23]. The risk of a major osteoporotic fracture was estimated using FRAX, OST, and SCORE, without involving BMD measurements. None of the strategies were substantially better than chance. It was concluded that fracture prediction requires risk factors not included in the current strategies. Therefore, the search for reliable prediction tools should continue [2].

Dental radiographs are among the most frequently made radiographs. Many studies of intraoral and panoramic radiographs report significant relationships with BMD and osteoporosis [24–31]. Studies with respect to the prediction of fractures are less abundant [32–36].

Lindh et al. developed an index to assess the trabecular pattern in periapical radiographs. Validated reference images from mandibular sections with characteristic trabecular patterns and typical distributions of trabecular bone were selected. With help of these reference images, observers assessed the trabecular pattern as dense trabeculation, alternating dense and sparse trabeculation, and sparse trabeculation [37]. The index was adapted by Lindh et al. and by Jonasson et al. [29, 33]. The assessment will be referred to as “observer score.”

The observer score of panoramic radiographs was used to predict postcranial fractures. For a group of 518 women, it was found that subjects with dense trabecular patterns had a hazard ratio of 0.07 for fractures in the following 26 years, whereas for subjects with sparse trabecular patterns, the hazard ratio was 3.63 [34]. The present study investigates if further improvement of fracture prediction can be obtained by texture analysis. Various texture analysis methods had been developed by Geraets and co-workers and by White and co-workers [10, 30, 31, 36, 38–44]. After selection of a region of interest (ROI) by an observer, these methods were applied automatically.

The main aim of the study was to maximize the predictive power of the trabecular pattern on panoramic radiographs. The secondary aim of the study was to parallel or even outperform the observer score with texture analysis methods.

## Materials and methods

### Subjects

The present study is based upon the Prospective Population Study of Women in Gothenburg, Sweden, a longitudinal study of perimenopausal women that had been randomly selected from the Revenue Office register. Participants gave their informed consent in accordance with the Helsinki Declaration. The study was approved by the Regional Ethical Review Board in Gothenburg (T453-04 and T075-09). Various medical and dental examinations were performed between 1968 and 2006 [34].

All women who had participated in the first part of the study were invited to also enter into the second part that started in 1980. Out of them, 73% underwent the medical and dental reexaminations. At the 1992 follow-up, an extensive non-participation analysis was performed. Non-participants were interviewed by means of a telephone call or a letter, and additional information was obtained from national registers and inpatient and outpatient records. Non-participants did not differ significantly from the participants except in long-term survival which was lower among the non-participants [45].

The present study focuses on women born in 1930 and 1922. In 1980, they were 50 or 58 years of age when a panoramic radiograph was made to assess the number of teeth, endodontic treatment, and the distance from the cemento-enamel junction to the bone crest. In 1992, the survivor participation rate was 69% for the medical examination and 64% for the dental examination [46]. At the end of the study in 2006, the subjects were 76 or 84 years of age. Then, the National Swedish Death Register was used to ascertain whether they were still alive. From the participating survivors, 304 women were selected randomly (*N* = 170 and *N* = 134 for age 76 and 84, respectively).

The occurrence of fractures between 1980 and 2006 was hospital-verified using the County Patient Register. No fractures of fingers and toes were recorded. Only clinical spine fractures were included. No attempt was made to separate fragility fractures from other fractures [34]. Women who sustained more than one fracture were included only once.

### Observer score and clinical variables

**Fig. 1:**
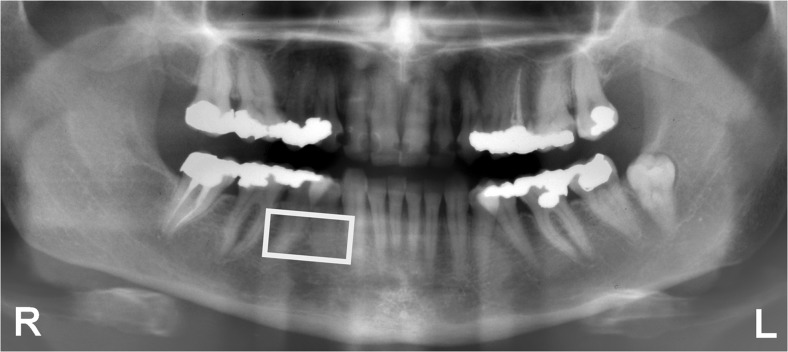
On the *right side* of the mandible, the area between the canine and the first molar was assessed as “sparse,” “sparse/dense,” or “dense” by one observer

Panoramic radiographs had been obtained during the 1980 examination with a Scanora (Orion Soredex, Helsinki, Finland) with 66–70 kV and 20 mA. In the present study, these radiographs were used to assess the trabecular pattern. They were placed on a light-box in a darkened room, and magnifying lenses (×2) were used. One observer (GJ), experienced in classifying the trabecular pattern in oral radiographs, closely inspected the trabecular pattern on the right side of the mandible between the canine and the first molar, at least 2 mm below the bone crest and at least 2 mm above the apices of the premolars (Fig. 1). Three radiographs were selected with characteristic trabecular patterns. A dense trabecular pattern has many well-mineralized trabeculae and small intertrabecular spaces. A sparse trabecular pattern has less trabeculae which are less-mineralized, and the intertrabecular spaces are mostly large. An alternating dense and sparse trabecular pattern is dense cervically and sparse apically. Densitometric measurements were performed to validate the reference radiographs [34].

With help of these reference radiographs, the radiographs were classified as dense, sparse, or alternating dense and sparse. In case of uncertainty, the category alternating dense and sparse was chosen. Crestal bone around teeth with marginal bone loss due to periodontitis was disregarded, as well as sclerotic bone around apices of problematic teeth. The assessment was blinded for fracture status.

A test-retest evaluation was done by the observer who had done the assessment described above and two other observers: an oral and maxillofacial radiologist and a general practitioner. They classified 30 panoramics twice 4 weeks apart [34].

In addition, the clinical variables age, weight, height, and BMI were recorded.

### Texture analysis

**Fig. 2:**
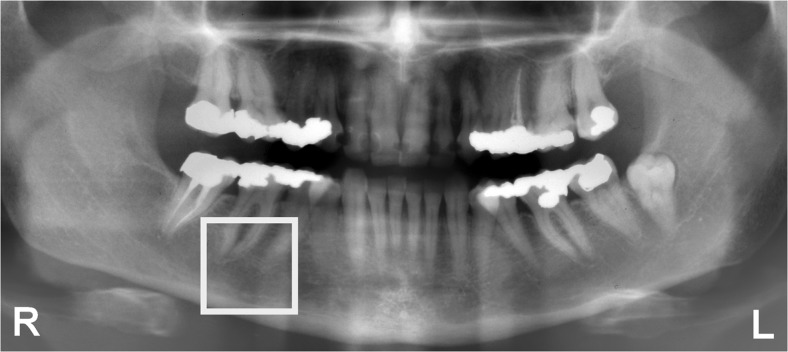
On the *right side* of the mandible, an ROI near the premolars was selected. The ROI measured 650 × 650 pixels and overlapped the area in Fig. 1

The radiographs were scanned with a flatbed scanner (Microtek Medi-2200 plus) at a resolution of 236 pixels per centimeter (600 dpi). First, an observer (WG) manually selected an ROI near the first molar and second premolar on the right side of the mandible. Afterwards, the ROI was adjusted automatically to a fixed size of 650 × 650 pixels, corresponding with 2.75 cm × 2.75 cm (Fig. 2).

The ROIs were subjected to automatic texture analysis procedures measuring various features that had proven their relevance for bone structure and osteoporosis [10, 25, 28, 30, 36, 39, 40, 42–44, 47–49].

**Fig. 3:**
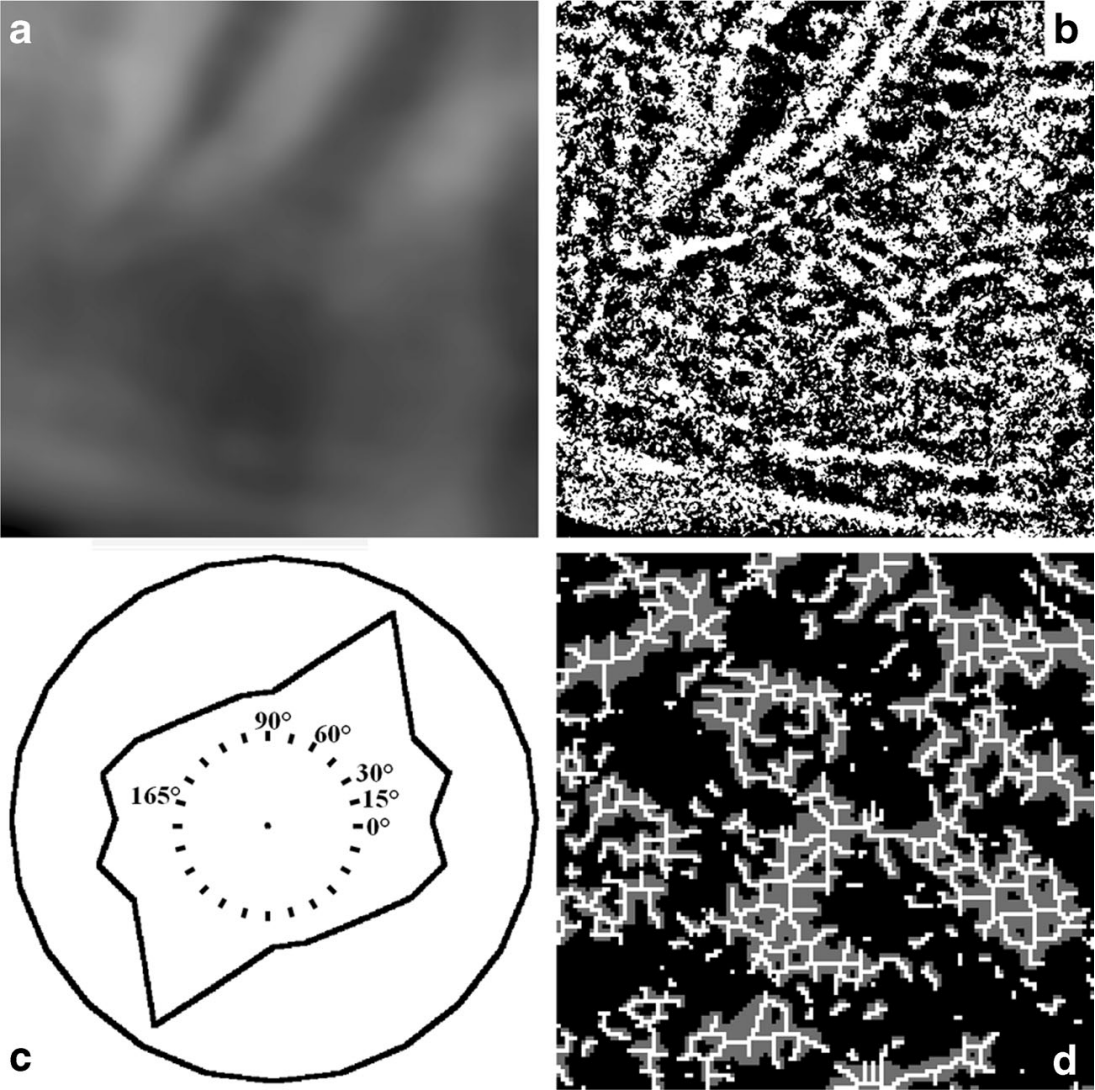
The ROI in Fig. 2 was subjected to texture analysis. **a** Blurred version of the ROI. To facilitate segmentation, the blurred version was subtracted from the original shown in Fig. 2. **b** Segmented ROI consisting of 650 × 650 pixels that are *black* if they belong to intertrabecular spaces, or *white* if they belong to trabeculae. **c** LFD orientation of the segmented ROI shown in **b**. Measurements were made along 0°, 15°,... and 165°. Along opposite directions, the LFD orientation is identical. This particular ROI has maximum LFD orientation along 60°. **d** Eroded ROI. To illustrate struts, nodes, and endpoints more clearly, the central part of 130 × 130 pixels has been taken and magnified 5 times. Eroded trabeculae are shown in *gray*. Struts are shown in *white*. Nodes have multiple white neighboring pixels, whereas endpoints have only one

First, brightness and contrast were determined. Then, a 3 × 3 median filter adjusted isolated pixels with deviating gray values. Next, an unsharp self-masking filter removed large-scale variations in gray value, caused by varying thickness of cortex and soft tissues (Fig. 3a). The sample was segmented into a binary image consisting of black and white segments (Fig. 3b).

The binary image was used to measure the number density of black and white segments, as well as their average area, perimeter, and width. The orientation of the binary image was measured in 12 directions (LFD_0°_, LFD_15°_,..., LFD_165°_) (Fig. 3c) [48]. Next, the white segments were eroded to quantify the average number of nodes and endpoints and the average strut length (Fig. 3d). Finally, the black segments were eroded and quantified in a similar way. The resulting measurements will be referred to as the “image features.”

### Statistics

Cohen’s Kappa-statistic was used to calculate the intra- and inter-observer agreement. This statistic was calculated with SAS version 9.2 (SAS Institute Inc., Cary, NC) [34]. Other statistical calculations were done with the SPSS package (version 21; SPSS Inc., Chicago, IL). To define significance, α = 0.05 was used.

Fracture patients were compared with patients that participated up to the end of the study and remained fracture free. *t* tests were applied to compare the two groups with respect to age, weight, height, and BMI.

The predictive power of three sets of variables was determined. The first set included the observer score and clinical variables age, weight, height, and BMI. The second set consisted of the image features that had been measured by the texture analysis procedures. The third set of variables combined the first two sets in order to test for any synergetic effect.

Forward stepwise logistic regression was applied to predict the occurrence of fractures. This analysis started with a prediction based on the prevalence of fracture patients and fracture free patients. Then, the variable was added that improved the prediction model most; this was repeated as long as a significant improvement was obtained. After completion of the prediction model, the corresponding ROC curve was constructed.

## Results

Of the 304 women, 115 sustained a fracture between 1980 and 2006. Fractures of the lower arm or wrist were most common (Table 1).

**Table 1:** Overview of fractures during 26 years of follow-up

304 women in total: 189 without fracture, 115 with fracture
8% had 1st fracture between 1980 and 1984
10% had 1st fracture between 1985 and 1989
15% had 1st fracture between 1990 and 1994
27% had 1st fracture between 1995 and 1999
23% had 1st fracture between 2000 and 2004
17% had 1st fracture between 2005 and 2006
35% lower arm or wrist
20% upper leg or hip
15% spine
12% upper arm
10% lower leg
9% other

One observer (GJ) assessed the trabecular patterns on the radiographs. Of the 304 subjects, 49 were classified as “dense,” 146 as “alternating dense and sparse,” and 109 as “sparse” (Table 2). For subjects with a sparse trabecular pattern, the odds ratio of sustaining a fracture was 11.6 (95% confidence interval (CI) 6.7–20.3). And if the pattern had been assessed as dense, the odds ratio of staying free from fractures was 38.8 (CI 5.3–285.5).

**Table 2:** Overview of observer scores of trabecular patterns

	Dense	Alternating dense and sparse	Sparse	Total
Fractured	1	35	79	115
Fracture free	48	111	30	189
Total	49	146	109	304

The Kappa value for the intra-observer agreement of observer GJ was 0.92. The Kappa values for the inter-observer agreement of observer GJ with the two other observers were 0.84 and 0.73, respectively, which indicates good agreement [34].

The descriptive statistics of age, weight, height, and BMI are provided in Table 3. Height was the only variable that differed for fracture patients and fracture free patients (*p* < 0.046). However, when taking into account that 4 variables were compared simultaneously, it was concluded that there was no significant difference between the two groups.

**Table 3:** Overview of clinical variables (mean ± SD): age in years, weight in kg, height in cm, BMI in kg/m^2^

	Age in 1980	Weight	Height	BMI
Fractured	54.3 ± 4.1	65.8 ± 9.9	164.4 ± 6.1	24.3 ± 3.4
Fracture free	53.9 ± 4.0	66.7 ± 11.0	163.0 ± 5.8	25.1 ± 3.7
Total	54.1 ± 4.0	66.3 ± 10.6	163.5 ± 5.9	24.8 ± 3.6

Logistic regression was applied on the observer score combined with age, weight, height, and BMI. The observer score was selected as the only predictor. This variable yielded an ROC curve with an AUC of 0.800. Age, weight, height, and BMI did not improve the prediction of fractures significantly (Table 4).

**Table 4:** AUC for prediction of fractures

	AUC	95% CI	Predictors
Observer score and clinical variables	0.800	0.749–0.851	Observer score
Image features	0.603	0.537–0.669	LFD45°
Combined	0.852	0.808–0.894	Observer score, LFD45°, contrast

Then, logistic regression was applied on the image features resulting in an ROC curve with AUC of 0.603 using the image feature LFD45°.

Finally, logistic regression was applied on the image features combined with observer score, age, weight, height, and BMI. The AUC was 0.852 using the observer score, LFD45°, and the contrast in the unfiltered ROI.

## Discussion

It is encouraging that the prediction based on the radiographic trabecular pattern yields an ROC curve with AUC 0.85 similar to the best prediction with AUC 0.84 using the Garvan algorithm [21]. The logistic regression analysis consistently selected the observer score as the most important predictor. If a sparse trabecular pattern was used as a predictor for fractures, then the prediction had a specificity of 84% and a sensitivity of 69%. These values define a point within distance 0.02 of the ROC curve described by Sandhu and co-workers [21]. Clearly, the observer score by itself can predict osteoporotic fractures nearly as accurately as the best prediction described in literature. Although the present study and the study by Sandhu et al. both are retrospective, they differ in the length of the follow-up, the age distribution, and the nationality of the populations. Therefore, the AUC values should be interpreted with caution.

The present study included only subjects that participated until the end of the study. Since non-participators tended to be less healthy than survivors, it is plausible that fractures and sparse trabecular patterns were more common among non-participators. So, any selection bias would probably not favor the prediction of fractures [50, 51]. Moreover, some of the fractures in this study may not have been fragility fractures. But there is evidence that the association with osteoporosis is similar for high- and low-trauma fractures [11].

A weak point of the present study is that the trabecular pattern was assessed by one observer (GJ) only. To some extent, that is compensated by the good agreement between the observer and two others. That makes it plausible that other observers would have assessed the trabecular patterns similarly. In addition, it is desirable to perform similar studies on other populations since the performance of fracture predictor tools varies over populations.

A strong point of the present study is the long fracture follow-up. The huge investment of time and effort needed for such studies explains why they are rare. Mostly, time is saved by predicting BMD values rather than the occurrence of fractures. However, since BMD values have low sensitivity for fracture prediction, they cannot replace long-term follow-up studies entirely.

In literature, there is renewed interest in cortical bone with respect to BMD and fragility [52–54]. Calciolari et al. show that the mandibular cortical width, the panoramic mandibular index, and the Klemetti index are overall useful panoramic measures to screen for low BMD. Yet, the relevance of such measures for postcranial fractures remains to be quantified. Zebaze et al. argue that the relevance of cortical bone with respect to bone fragility has been neglected. They show that in the aging distal radius, the amount of cortical bone loss doubles the amount of trabecular bone loss [54]. Moreover, they show that in the aging femur, the remodeling surface in cortical bone exceeds the remodeling area in trabecular bone. At the age of 29, the pores in the cortex are small spheres of about 0.07 mm diameter, evenly distributed throughout the cortex. Such pores in the mandibular cortex would be invisible on panoramic radiographs since even the best panoramic devices resolve details of 0.1 mm minimum. However, at the age of 67, the pores have increased in size up to 0.5 mm and their shape is irregular. At the age of 90, most of the femoral cortex has been trabecularized. If the mandibular cortex degrades in a similar way, then it is plausible that the cortex contributes to the radiographic trabecular pattern. For intraoral radiographs, this holds even more since they resolve smaller details throughout the mandibular bone. The phenomenon that cortical bone gradually becomes trabecularized implies that any technique to distinguish between cortical and trabecular bone can be questioned. It should be realized that the trabecular pattern not necessarily originates from trabecular bone only.

Panoramic radiographs have various geometric distorsions and provide less details than intraoral radiographs. Proper positioning of the patient is necessary to obtain a useful panoramic radiograph. Therefore, panoramic radiographs are harder to assess than intraoral radiographs, and assessing panoramic radiographs requires extra training. The intra- and inter-observer agreement for panoramics is lower [55]. However, intraoral radiographs were not included in the original Prospective Population Study of Women in Gothenburg.

Moreover, it might be that the situation is different from the viewpoint of texture analysis. In a previous study, the texture analysis methods were applied on panoramic radiographs as well as on intraoral radiographs [10]. The measurements were used to predict total hip BMD and spinal BMD. It was found that panoramic radiographs on average contributed more to the predictions than intraoral radiographs of the mandible and maxilla. A related study used the texture analysis methods to predict osteoporosis [30]. After age, the second most important predictor originated from the panoramic radiographs whereas the next most important predictor originated from intraoral radiographs of the mandible. This demonstrates that panoramic radiographs may be more important than intraoral radiographs when it comes to predicting BMD and or osteoporosis.

It is shown that the trabecular pattern on panoramic radiographs contains important clues for predicting postcranial fractures. These clues are picked up by the human observer and to a lesser extent by the texture analysis procedures even though image features like width and area of the “trabeculae” and “marrow spaces” had been designed to quantify the coarseness of the trabecular pattern. This may be due to the knowledge that an experienced dentists has of the human anatomy.

## Conclusion

Conclusively, it can be said that the observer score of the trabecular pattern is a sophisticated evaluation not yet achieved by machine analysis. The present study demonstrates that the radiographic trabecular pattern contains information for a state of the art prediction of future fractures, at least in postmenopausal women. Considering the social burden of fractures and the low costs involved with panoramic radiographs, further study of the radiographic trabecular pattern is justified.
